# Seeing It All: Evaluating Supervised Machine Learning Methods for the Classification of Diverse Otariid Behaviours

**DOI:** 10.1371/journal.pone.0166898

**Published:** 2016-12-21

**Authors:** Monique A. Ladds, Adam P. Thompson, David J. Slip, David P. Hocking, Robert G. Harcourt

**Affiliations:** 1 Marine Predator Research Group, Department of Biological Sciences, Macquarie University, North Ryde, New South Wales, Australia; 2 Digital Network, Australian Broadcasting Corporation (ABC), Sydney, New South Wales, Australia; 3 Taronga Conservation Society Australia, Bradley's Head Road, Mosman, New South Wales, Australia; 4 School of Biological Sciences, Monash University, Melbourne, Australia; 5 Geosciences, Museum Victoria, Melbourne, Australia; Nagoya University, JAPAN

## Abstract

Constructing activity budgets for marine animals when they are at sea and cannot be directly observed is challenging, but recent advances in bio-logging technology offer solutions to this problem. Accelerometers can potentially identify a wide range of behaviours for animals based on unique patterns of acceleration. However, when analysing data derived from accelerometers, there are many statistical techniques available which when applied to different data sets produce different classification accuracies. We investigated a selection of supervised machine learning methods for interpreting behavioural data from captive otariids (fur seals and sea lions). We conducted controlled experiments with 12 seals, where their behaviours were filmed while they were wearing 3-axis accelerometers. From video we identified 26 behaviours that could be grouped into one of four categories (foraging, resting, travelling and grooming) representing key behaviour states for wild seals. We used data from 10 seals to train four predictive classification models: stochastic gradient boosting (GBM), random forests, support vector machine using four different kernels and a baseline model: penalised logistic regression. We then took the best parameters from each model and cross-validated the results on the two seals unseen so far. We also investigated the influence of feature statistics (describing some characteristic of the seal), testing the models both with and without these. Cross-validation accuracies were lower than training accuracy, but the SVM with a polynomial kernel was still able to classify seal behaviour with high accuracy (>70%). Adding feature statistics improved accuracies across all models tested. Most categories of behaviour -resting, grooming and feeding—were all predicted with reasonable accuracy (52–81%) by the SVM while travelling was poorly categorised (31–41%). These results show that model selection is important when classifying behaviour and that by using animal characteristics we can strengthen the overall accuracy.

## Introduction

Advances in bio-logging technologies have provided a means by which we can accurately quantify the activity budgets of marine predators [1, 2]. Previously, investigators have used multiple devices and/or direct observation to investigate a single parameter [e.g. feeding; 3, 4]. Observation allows researchers to record detailed behaviour without directly interacting with the animal, though this method is often inefficient due to the inability of researchers to record behaviour at all times and is biased to observations at or near the surface [5]. In addition, marine predators are difficult if not impossible to observe in the wild as they spend most of their time underwater and can forage over great distances [1]. Well documented observer effects add to the limitations of direct observation, and this has lead researchers to develop devices that allow us to record animal behaviour remotely [6].

Time-depth recorders and stomach temperature loggers have been used in combination to predict when an animal has captured and ingested prey [7]. However, gaining complete information from a multi-instrument approach can be invasive, expensive, analytically complicated and is not always successful [8]. A more refined approach is to use devices that can measure physical activity over periods long enough to be representative of typical daily activities, with minimal discomfort to the animals, and applicable to large populations [9]. Tri-axial accelerometers are one option, as these can measure animals in their natural environments over long periods and in places where observation is difficult or impossible [1, 10]. These devices are increasing in popularity and offer opportunity to study marine predators with a level of detail that other devices do not [11]. They allow us to measure and classify the activity of animals using data from a single device [12], and can be incorporated into more complex devices along with sensors that record physical and environmental parameters such as depth and temperature [13]. Unique combinations of the three accelerometry axes; heave, surge and sway, can be used to identify different activities [11]. Feeding events can be identified from mandible and head mounted accelerometers [3, 14, 15], but a wider range of behaviours, and a proxy for the energy expenditure of those behaviours, may be predicted from mounting the device close to the mid-point of the animals torso [16].

Currently many methods and techniques exist for the classification of accelerometry data. Supervised and unsupervised algorithms provide options for classification and interpretation [14, 17]. Supervised learning can adjust its classifications by using error messages programmed by the user, whereas unsupervised learning looks for patterns in the data. Supervised learning requires the input of a ‘teacher’ to manually classify the behaviour and to ‘teach’ the program how to identify each behaviour [18]. This method can be highly accurate and precise, but is also very time consuming. In contrast, unsupervised learning classifies behaviour using heuristics [18]. Unsupervised learning has the advantage of speed, trading it for accuracy or precision. It may also be able to pick up patterns in the data that manual classification methods do not. When classifying data for supervised learning there is a degree of subjectivity involved on behalf of the teacher, whereas unsupervised learning algorithms classify data with an unbiased view [6].

Published ethograms have used a wide variety of these methods with varying degrees of success, including quadratic discriminant analysis (QDA) for the classification of activity in cattle and humans [19], decision trees with turtles [20] random forests with badgers [21, 22], and neural networks with humans [23]. Each method has advantages and disadvantages, and it is likely that different methods will work better for different species, device placement and settings. With the significant advancement of computer speed and the relative ease with which these methods can be implemented an important step is to determine the most appropriate method of analysis for the particular set of circumstances under study.

To explore this, we used data from captive otariid pinnipeds to assess the reliability of a number of different machine learning algorithms in identifying particular behaviours. Activity budgets of otariids include activity on land and in water, and water behaviours can be more complex to define as they involve dynamic movement in a 3D environment. To date, quantifying pinniped behaviour using accelerometers has focussed on identifying foraging and travelling behavioural states [24]. Less attention has been paid to other potentially important behaviour states, such as grooming, reproductive and resting behaviours, despite these being major components of their behavioural repertoire and possible indicators of important underlying indicators such as condition [25, 26]. As yet, no studies have sought to quantify the terrestrial behaviours displayed by pinnipeds using accelerometers. The aims of this paper were (1) to build a detailed ethogram of the key behaviours performed by captive otariid pinnipeds, applicable to wild populations, and (2) to use a range of machine learning algorithms to classify these behaviours, providing us with the opportunity to test and compare the accuracy of these different methods.

## Materials and Methods

### Animals

We conducted experiments with two Australian fur seals (*Arctocephalus pusillus doriferus*), three New Zealand fur seals (*Arctocephalus forsteri*), one subantarctic fur seal (*Arctocephalus tropicalis*), and six Australian sea lions (*Neophoca cinerea*) (Table 1), from three Australian marine facilities: Dolphin Marine Magic, Coffs Harbour (RF1: -30°17’N, 153°8’E); Underwater World, Sunshine Coast (RF2: -25°40’N, 153°7’E); and Taronga Zoo, Sydney (RF3: -33°50’N, 151°14’E). Experiments were conducted from August to November 2014 at all three institutions, and again in August 2015 at RF2. The seals were on permanent display at their respective marine facilities and were fed and cared for under the guidelines of the individual facility. All Australian sea lions in the study were born as a part of an ongoing captive breeding program in Australian aquaria, while all fur seals came into captivity as juveniles, in poor health or injured, and were considered unsuitable for release. All fur seals were in very good health during the study. This study was conducted under permits from Macquarie University ethics committee (ARA-2012_064) and Taronga zoo ethics committee (4c/10/13).

**Table 1:** Identification number, location, species, age weight and sex of seals with number of sessions and attachment method of accelerometer. AFS—Australian fur seal; NZFS—New Zealand fur seal; SFS—subantarctic fur seal and ASL—Australian sea lion.

Seal ID	Marine facility	Species	Age	Weight range (kg)	Sex	Number of sessions	Attachment method
ASF1	RF1	ASL	5	44–47	Female	13	Harness
ASF3	RF2	ASL	17	58–74	Female	4	Harness
ASF4	RF1	ASL	17	66–70	Female	12	Harness
ASF6	RF1	ASL	7	50	Female	2	Harness
ASM1	RF1	ASL	9	108–110	Male	8	Harness
AFF1	RF2	AFS	17	69–79	Female	7	Tape
AFM1	RF2	AFS	16	175–242	Male	7	Tape
ASM2	RF3	ASL	13	160–162	Male	9	Tape
NFM1	RF3	NZFS	8	47–54	Male	5	Tape
NFM2	RF2	NZFS	11	108–152	Male	5	Tape
NFM3	RF3	NZFS	13	111–154	Male	8	Tape
SFM1	RF2	SFS	4	28–30	Male	3	Tape

### Experimental protocol

Seals were fitted with a tri-axial accelerometer (CEFAS G6a+: 40mm x 28 mm x 16.3 mm,18 g in air and 4.3 g in seawater, CEFAS technology Ltd, Lowestoft, UK) positioned between the shoulder blades. Accelerometers recorded three axes of acceleration: surge (x-axis), sway (y-axis) and heave (z-axis). They were orientated such that the x-axis was anterior–posterior, the y axis was lateral and the z axis was dorsal–ventral. Accelerometers recorded at +-8g, at a rate of 25 samples per second (25Hz), and logged wet/dry events.

**Fig 1:**
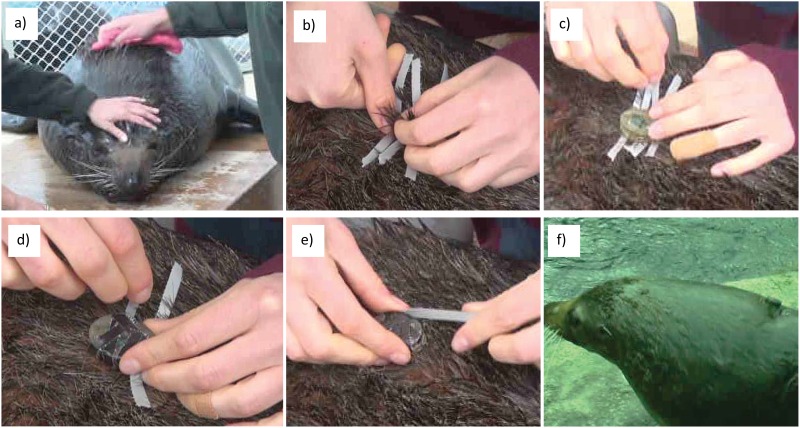
Process of accelerometer attachment with tape. a) Dry the fur; b) Lift the hair to stick tape to undercoat; c-e) Tape on the accelerometer; f) Seal with accelerometer.

**Fig 2:**
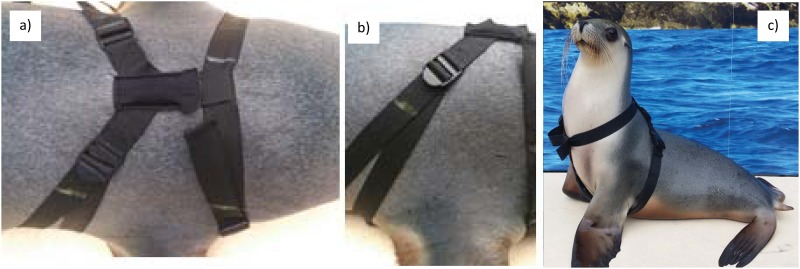
Harness. a) Back; b) Side; c) Front.

For fur seals accelerometers were secured between the shoulder blades on the top layer of fur using Tesa tape (Tesa, Eastern Creek, NSW, Australia; Fig 1). The process took around 2 minutes to attach and 30–60 seconds to remove. This method could not be used for the sea lions as the fur was too short for the tape to hold the devices. Instead, we used a custom designed harness ((c) Guy Bedford) with three clips, one around the neck and two at the back (Fig 2), and accelerometers were fitted into a pocket sewn to the back.

Each session was recorded using two or three cameras filming at 50 frames per second (FPS); one or two cameras (GoPro Hero 3 –Black edition, USA) were placed in a pool below the water line to capture all underwater behaviour and above water behaviour was captured by a hand held camera (HDRSR11E: Sony, Japan). Depending on the seal and the facility we were working at we altered the pools that we were using. At RF1 we used three pools, the first pool was 11m diameter and 3m deep, the second pool was 12m wide, 24m long and an average depth of 2m, the third pool was 7m diameter and 2m deep. At RF2 we used one large pool which was 11m wide, 14m long with an average depth of 8m. At RF3 we used three pools, the first was 6m wide, 15m long and an average of 3m deep, the second pool was 9m wide, 12m long and an average of 3m deep, the third pool was 26m long, 9m wide and 5m deep. We defined a session as a continuous period that seals were wearing the accelerometer and being filmed, and we attempted only one session per day per seal. Sessions had a maximum duration of 90 minutes after which the accelerometer removed and the seal was rewarded. Seals participated in 3–11 sessions.

We observed seals during training sessions where behaviours were requested using operant-conditioning, and also without conditions. Seals were not restrained or required to give a behaviour. We observed two types of sessions; feeding and behaviour sessions. The feeding sessions aimed to provide seals with large food items that required some form of processing prior to eating [see 27]. Seals were given a range of seafood including bream (*Abramis brama*), mullet (*Mugil cephalus*), Sydney octopus (*Octopus tetricus*), Australian salmon (*Arripis truttaceus*), mackerel tuna (*Euthynnus affinis*), New Zealand brill (*Colistium guntheri*) and yellowtail amberjack (*Seriola lalandi*). Seals entered the water and were given the particular food item in the water with an unrestricted amount of time to eat. When a seal did not eat the food either another seal was introduced to the pool to encourage competition, or the original seal was returned to its pen and a different seal was fitted with an accelerometer and presented with the food.

Behaviour sessions also incorporated some feeding events with small fish that did not require processing. Fish were thrown in the pool so that seals had to capture them mid-water as they sank. During each behaviour session seals were instructed to perform a series of natural behaviours from their known behavioural repertoire (S1 File). These behaviours were expected to emulate the behaviour of wild seals, such as porpoising, swimming and grooming. Behaviours were repeated during a session until the food was exhausted or the seal did not respond to instruction.

### Statistical analyses

#### Data preparation

The acceleration data were downloaded using the G5 Host software (Version 6.4 CEFAS Technology Ltd). The video from each camera was imported into Adobe Premiere Pro CC (Adobe Systems Inc., California) where it was synced so that the video files could be easily viewed together. They were then exported at 25 FPS as a single movie file. Data were coded manually using Excel (Microsoft Corp., Washington, USA) and Quicktime (Apple Computer Inc., California, USA). To synchronise the accelerometer and the video, we “marked” the accelerometer on the video by hitting it against a hard surface while filming. This caused a large spike in the accelerometry data that we could match exactly to the video. We matched each accelerometry data sample with the corresponding video frame and the specific behaviour recorded in Excel (see S1 File for a detailed list of behaviours and their descriptions). Videos were scored without interruption.

The duration of a behaviour ranged from 0.25 (e.g. shake) to 3.5 minutes (e.g. continuous swimming). We coded 26 unique behaviours, but because there were not enough samples of each of the individual behaviours, we grouped behaviours into five categories. These behaviour categories were chosen based on a combination of ecological and behavioural knowledge of the target species, rather than on statistically identifiable behaviours (as in unsupervised learning). The five categories were grooming, travelling, foraging, resting and other. The ‘other’ category consisted of direct feeding by the trainer (when the food was delivered by hand or thrown and caught), behaviours that could not be clearly placed into one of the other categories, and time where the seal was out of sight. As these cannot be considered natural behaviours, accelerometry data collected at these times was not included in the analysis. Where behaviours overlapped, or were displayed simultaneously (e.g. foraging and travelling), grooming and foraging took precedent over travelling and resting. Half of the videos were coded by two coders (JK and ML) and compared for validation. The coders recorded the same behaviour in over 95% of cases, therefore the first coder (JK) completed the remaining coding.

Data were summarised into epochs (sliding sample windows) of length 13 which represented approximately 0.5 sec data. This would ensure that the shortest recorded behaviour would be captured. Data were further split into training and testing, where ten seals data were used for training and two seals data were kept for cross-validation of the models. One female sea lion and one male fur seal were selected which represents the range of animals in our dataset.

#### Summary statistics

Choosing the number of summary statistics that are put into a model can be highly subjective. Complex behaviours, and large numbers of example behaviours means that a large number of summary statistics are likely required. A greater number of summary statistics improves the algorithms chances of detecting subtle differences between the behaviours [6, 28]. We coded 52 summary statistics and added five feature statistics describing some characteristic of the individual or the event to the second stage of model testing. These were included to assess their overall impact on prediction performance of the models. The features we included were device attachment method (harness or tape), age, mass, sex and species of the individual. We included where the behaviour occurred (surface, underwater or land) in all models. We calculated summary statistics including mean, median, standard deviation, skewness, kurtosis, minimum, maximum, absolute value, inverse covariance, autocorrelation trend (the coefficient derived from a linear regression) for each of the three axes. We also calculated *q* as the square-root of the sum-of-squares of the three axis [17], and included pair-wise correlations of the three axis (x-y, y-z, x-z) [29]. The inclination as azimuth were calculated as per Nathan et al. [17]. We calculated three measures of dynamic body acceleration (DBA) by first using a running mean of each axis over 3 seconds to create a value for static acceleration. We then subtracted the static acceleration at each point from the raw acceleration value to create a value for partial dynamic body acceleration (PDBA). The values of PDBA on each axis were summed to calculate overall dynamic body acceleration (ODBA; Eq 1) [30, 31]. We calculated vectorial dynamic body acceleration (VeDBA; Eq 2) as the square root of the squared PDBA of the three axis [32] and calculated the area under the curve for both ODBA and VeDBA using the package “MESS” in R [33, 34].

$$ODBA=\;\left|X_{dyn}\right|+\left|Y_{dyn}\right|+\left|Z_{dyn}\right|$$ (1)

$$VeDBA=\;\sqrt{X_{dyn}^{2}+Y_{dyn}^{2}+Z_{dyn}^{2}}$$ (2)

#### Penalised logistic regression

In logistic regression the probability of each outcome was estimated via a logistic function which transformed a binary [0, 1] outcome to a continuous outcome from negative infinity to positive infinity. A linear relationship was then found between the transformed outcome and the input variables (this process was performed in one step, but is easier to visualise as a two stage process). A penalty was added to the error function of this process to avoid over fitting of the problem. Common forms of this penalty are either the L1 or L2 norm. In effect this penalty shrinks the coefficients of the logistic regression towards zero, to simplify the model. We implemented logistic regression to set a base line accuracy against which the other, more complicated models were compared. The penalised logistic regression was implemented using the R package “glmnet” [35].

#### Support vector machines

Support vector machines (SVM) are a form of discriminant classifier, where this discrimination was performed by hyperplanes that divide the input data into classes according to their labels [36]. In essence two hyperplanes were employed and the distance between them chosen to maximise the distance between the two classes. Hence a SVM is often referred to as a maximal margin classifier. The simplest form of SVM used a linear kernel to find a way to linearly separate the classes. Often the data do not separate linearly in which case nonlinear kernels were used to map the features to different vector spaces where it may be possible to better separate the data. We tested linear, polynomial, radial and sigmoid kernels. The SVM was implemented using the R package “e1071” [37].

#### Random forests

Random forests are a form of ensemble learning [38]. An ensemble is a combination of different classifiers (referred to as base learners) each trained to perform the same classification, generally in a slightly different way, then the results are combined (generally averaged) to give the final output. In a random forest the base learners are decision trees. Decision trees attempt to partition the feature space one variable at a time in the way that best classifies the data (i.e. the input variables are divided such that values above a point go into one class and values below a point go into a different class). This partitioning (splitting) of the input variables continues until no more splits can be performed or some stopping criteria are reached. To create a random forest, many decision trees were trained with each tree only seeing a random subset of the data, and at each split a random subsample of the input variables was tested for partitioning. Finally, all of the trees were averaged to generate output probabilities. The random forest was implemented using the R package “randomForest” [39]

#### Stochastic gradient boosting

Stochastic gradient boosting machines (GBMs) are another form of ensemble learning [40]. Although base learners can be in many forms, we implemented tree learners as the base learners. GBMs pre-form classification in an iterative fashion. In the first iteration a learner is trained to classify the problem. In each successive iteration another base learner is trained to explain the error from the previous iteration. Thus a GBM successively learns to explain the error of all previous iterations. Iterations continue until a stopping criterion is reached, generally the maximum number of iterations. GBMs are stochastic in nature due to each iteration is only shown a randomly selected subset of the data and at each stage in the tree building process only a random subset of the input variables is assessed for splitting. To generate output probabilities all of the trees were averaged. The GBM was implemented using the R package “xgboost” [41]

All models were run in R (version 3.2.1) through the package “caret” [42].

#### Training and testing

The data classes were imbalanced, therefore the effects of both under and over-sampling were tested and the resulting model performance assessed. Over-sampling can cause the model to over-fit, whereas under-sampling may lose vital information [43]. Initial testing showed that under-sampling performed slightly better than over-sampling, therefore under-sampling was used for the rest of the testing. Moreover, due to the large amount of data that we had under-sampling was used with little restriction. We chose a class maximum to be 3000, smaller than the minority class size of 4084. Under-sampling was only used for the training data. Test data was left unchanged as it was more representative of wild data that would not be evenly distributed among behaviour groups.

In order to assess the influence of the feature statistics on our models we ran each model twice, once with the summary statistics and once with the feature and summary statistics. To find the best parameters of the models the data with ten seals were split into training and validation sets, which were 70% and 30% of the data respectively and run across a grid of parameters. The models were trained on the (70%) data split using 10-fold cross validation. Model performance for the data is as an average of the out of fold accuracy, e.g. the model is trained on 9-folds and then tested on the 10^th^ fold. This process was repeated 10 times, each time using a different fold as the out-of-sample data, until all folds had been used. The final model performance (reported here) was the accuracy on the 30% validation split from which we found the best parameters for each model. We used these parameters to train a model with the data from the ten seals and used it to classify the behaviours of the two seals that were so far unseen by any model. Thus the final cross-validation accuracy was assessed on data that the model had not seen during training and gave a true picture of model generalisation.

## Results

**Fig 3:**
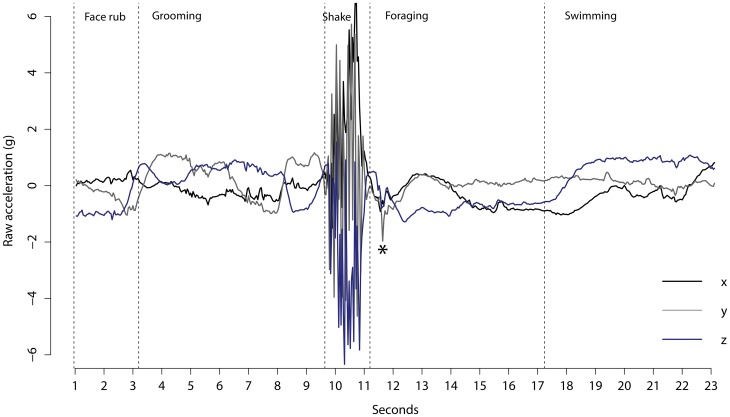
Example of raw acceleration data for a series of behaviours. The * represents a fish capture in the water column.

Through coding more than 20 hours of video footage we classified 5817 bouts split between the 27 behaviours (Table 2). Bouts of behavioural were clearly identifies from the tri-axial accelerometry data (Fig 3). 1344 bouts of behaviour were classified as other because they were behaviours that would not be seen in the wild (i.e. moving in and out of the pool, being fed by the marine mammal keeper) and were excluded from the analysis. This included 30 bouts of behaviour classified as playing, and while this behaviour in the wild is an important indicator of development and condition [44, 45] the sample size was too small to compare it to the other groups of behaviour.

**Table 2:** Number of bouts of behaviours classified and their associated categories.

Category	Behaviour	Number of bouts	Category	Behaviour	Number of bouts
Travelling (N = 2844)	Walking	535	Resting (N = 883)	Lying	17
	Surface swimming	1128		Sitting	532
	Swimming	1003		Still	280
	Fast	121	Grooming (N = 331)	Scratch	67
	Porpoising	57		Rubbing	9
Feeding (N = 1759)	Chewing	308		Sailing	28
	Searching	249		Jugging	19
	Thrash	303		Face rub	54
	Manipulation	779		Shake	39
	Hold and tear	120		Rolling	115

Using 13 epochs we had a total of 92516 input variables for the model. This consisted of 64642 training inputs and 24795 testing inputs from the two seals selected for cross-validation. The final average accuracy from the training set of data without feature data for the baseline model (penalised logistic regression) was 64.0%, with poor testing results (47.0%). From the training results without features random forests were the most accurate in predicting behaviours, classifying on average 75.1% of the behaviours accurately (Table 3). However, the cross-validation accuracy for this model was poor (48.6%). This was followed by stochastic gradient boosting machines (GBM) with an average accuracy of 73.7%, with cross-validation accuracy of 62.0%. SVM’s achieved between 64.2 and 72.6% accuracy, with cross-validation scores ranging from 48.0 to 64.0%. The kernel used for SVM’s was important in determining final accuracy where linear kernels produced the lowest accuracies and polynomial kernels produced the highest accuracies overall (Table 3).

**Table 3:** Average training (ten animals) and testing (two unseen animals) accuracy of machine learning models run with and without feature statistics and the best parameters used for testing.

Model	Train Accuracy	Test Accuracy	Best parameters
Features = FALSE			
GBM	73.69	61.98	Eta = 0.01; max.depth = 5; nrounds = 5000; subsample = 0.7
RF	75.08	48.63	Mtry = 10; ntree = 1400, nodesize = 1
RLR	63.72	46.91	Param1 = 0.810 param2 = 0.0012
SVM Linear	64.22	48.00	Cost = 100
SVM Sigmoid	65.08	46.29	Gamma = 0.0001; coef0 = 0; cost = 100
SVM Radial	71.25	59.71	Gamma = 0.001; cost = 100000
SVM Polynomial	72.58	63.94	Degree = 4; gamma = 0.01; coef0 = 4; cost = 1
Features = TRUE			
GBM	80.81	65.04	Eta = 0.01; max.depth = 4; nrounds = 5000; subsample = 0.8
RF	80.53	53.92	Mtry = 12; ntree = 1000, nodesize = 3
RLR	71.33	64.63	Param1 = 0.10 param2 = 0.0018
SVM Linear	71.50	68.15	Cost = 10
SVM Sigmoid	70.31	55.46	Cost = 100; coef0 = 0; gamma = 0.0001
SVM Radial	79.03	68.87	Cost = 10000; gamma = 0.001
SVM Polynomial	78.83	72.01	Cost = 0.1; coef0 = 4; gamma = 0.01; degree = 4

Adding feature data to the models improved the training and testing accuracy of all models. Random forests and GBM achieved over 80% training accuracy, though GBM had better performance on cross-validation (65.0%) than random forests (54.0%). Despite having lower training accuracy than the GBM and random forest, the SVM with polynomial, linear and radial kernels all had higher cross-validation accuracies. The polynomial kernel had the highest cross-validation accuracy of any model, classifying 72.0% of the data accurately.

Within the training models resting was most often classified accurately (83–89%), followed by grooming (71–94%) and foraging (59–75%). Travelling was the most difficult category to classify (32–71%) (Table 3). The confusion matrices for the cross-validation accuracies on the two seals left out reveal a very different story and model influenced the overall accuracy of each behaviour category (Table 4). Travelling was still the hardest behaviour to classify (31–58%) and the models now found resting much harder to classify (41–75%). Foraging was able to be classified with the highest accuracy now (60–85%) followed by grooming (62–76%).

**Table 4:** Confusion matrix for the cross-validation results from the GBM, RF, LR and SVM models. ^Only the results from the best SVM (polynomial) are presented here.

**GBM**	**Foraging**	**Grooming**	**Resting**	**Travelling**	**Sensitivity**	**Specificity**
**Foraging**	5717	66	132	821	84.9%	88.3%
**Grooming**	42	180	10	59	61.9%	71.4%
**Resting**	363	66	1773	332	70.0%	70.2%
**Travelling**	2226	1111	5020	11397	57.7%	36.0%
**RF**	Foraging	Grooming	Resting	Travelling	Sensitivity	Specificity
**Foraging**	4836	661	257	982	71.8%	74.9%
**Grooming**	36	183	16	56	62.9%	61.9%
**Resting**	508	38	1830	158	72.2%	60.2%
**Travelling**	3996	3681	1037	6520	42.8%	43.7%
**LR**	Foraging	Grooming	Resting	Travelling	Sensitivity	Specificity
**Foraging**	5671	115	174	776	84.2%	80.3%
**Grooming**	14	202	21	54	69.4%	62.4%
**Resting**	441	47	1843	203	72.7%	60.6%
**Travelling**	3094	3024	806	8310	54.5%	35.9%
**SVM**	Foraging	Grooming	Resting	Travelling	Sensitivity	Specificity
**Foraging**	5856	123	62	695	86.9%	81.3%
**Grooming**	52	188	6	45	64.6%	62.5%
**Resting**	697	314	1040	483	41.0%	61.6%
**Travelling**	2596	1258	483	10772	71.3%	30.9%

## Discussion

Accelerometers have been used to build ethograms in a range of species, generally being able to predict the correct classification of a class more than 90% of the time, however we argue that this may be a result of highly selective data input and choices made in the analysis. In this study, we trained machine learning models to recognise four distinct, biologically-relevant, categories of behaviour: travelling, resting, foraging and grooming. Models were then tested on two seals previously unseen by the models and were tested both with and without feature statistics describing some characteristic of the seal. The choice of machine learning algorithm contributed to the overall prediction accuracy and adding feature statistics to the model improved the overall training and testing accuracies. By training our models on all seals and testing two left out we are ensuring the generalisability of our models and that they are robust to individual differences.

### Supervised machine learning

Machine learning algorithms have regularly been used to classify animal behaviour from accelerometry data, with varying levels of success [10, 20, 46]. With a range of algorithms available and the wide array of problems to which they can be applied, it can be overwhelming to be able to select an appropriate method that will provide the greatest accuracy [17]. Rapidly developing technology has improved computing speed and the ease by which machine learning can be implemented. This affords researchers the opportunity to test and examine different methods for their data. Here we tested four supervised machine learning algorithms on accelerometry data collected from captive fur seals and sea lions to assess their ability to predict behavioural states. We found that SVM with a polynomial kernel was the most accurate in being able to classify behaviours from testing data (previously unseen by the model), but that GBM and random forests produced the best training results.

In a study on the behavioural modes of griffon vultures (*Gyps fulvus*) five machine learning algorithms were evaluated with random forests being the best predictor of behaviour [17]. While random forests also performed well when evaluating training data in our comparison, GBM (which was not evaluated by [17]) improved the accuracy. However, SVM with a polynomial kernel had the highest rate of cross-validation classification accuracy. SVM’s have been used successfully in other behaviour classification studies that used accelerometers [47–49]. It is likely that the best classification algorithm will differ for each data set and the behaviour type that is to be predicted. We found that different machine learning algorithms gave better results depending on whether it was training or testing the data. They also differed in the accuracies assigned to different behaviour categories. Given the large variety of machine learning algorithms available and the relative ease of implementation and testing, we recommend evaluating a range of different algorithms to determine which gives the best performance for a particular problem.

### Groups of behaviours

We classified 26 behavioural states (S1 File), one of which (playing), was not used as it occurred infrequently. This was too many groups for a model to classify realistically in terms of computational time and power. It also required a large investment of observer time in order to collect a large enough sample for each of the classes represented in the model. This is because an important step in the process is to ensure each behaviour or class is equally represented in the model. Rather than losing the detailed information of each of the observed behaviours, we grouped behaviours into states [e.g. 50]. This technique can be useful in developing activity budgets for large data sets, particularly where one state dominates behaviour (e.g. swimming). This method may also prove useful in wild applications that aim to automatically classify the state of the animal in real time, before uploading a wireless data summary to a nearby receiver. Summarised data from accelerometers via wireless devices have been successfully used for monitoring human behaviours [51], in particular for monitoring health conditions [52, 53], but have not as yet been used for monitoring wild animals. This advance in technology has the potential to increase the efficiency and the data storage capacity of devices on wild tagged animals.

The four categories we created for this analysis (grooming, resting, travelling, foraging), represent the typical behaviours that would be used by these species in the wild [54, 55]. Resting had fewest cases of misclassification in the training stage as there was minimal movement on any axis and was consequently easy to predict. However, in the testing stage the prediction accuracy of resting, while still reasonable dropped 10–30% depending on the model. The models predicted grooming with reasonable accuracy in both training and testing which was probably from using a relatively short epoch allowed more active behaviours to be distinguished from immobile behaviours [56]. Travelling was predicted with the least accuracy in training and testing. Travelling was most commonly mistaken for foraging, which is not surprising considering the behaviours frequently overlapped. Foraging was predicted well, likely at the detriment of travelling. Usually, foraging behaviours are the most difficult to distinguish, particularly when they are of very short duration (such as a fish capture here or attack/peck in the plover [57]). Having a very short epoch likely allowed these behaviours to become more distinguished, while travelling behaviours became nosier. Repetitive behaviours perform better with longer epochs as the model is more readily able to find the patterns in the data [48]. Therefore using a longer epoch will likely strengthen the models ability to predict resting and travelling, but will reduce the accuracy of grooming and foraging.

There are some obvious categories of behaviour fundamental to the ecology of fur seals and sea lions that we were unable to capture. Play behaviour is an indicator of developmental stage and also a subtle indicator of changes in condition [44, 45], but we had insufficient samples for analysis. Mating and social behaviours are largely absent from the accelerometry literature [6], and here we were unable to fill this gap as we did not record the animals mating. Because it is inherently difficult to observe mating behaviour, accelerometers have only been used for identifying reproductive behaviour of free-living animals in a few instances [58]. Other behaviours that we did not observe but are known to be important in otariid ecology include regurgitation and vocalisations [59]. The absence of these behaviours from this ethogram means that when these behaviours are captured in the wild, the learning algorithm will classify these as one of the pre-determined categories on which we have trained the model. When monitoring an animal over an extended period it can result in a misrepresentation of how animals spend their time.

### Leave-two-out validation methodology

A goal of this study is to be able to generate a robust model that can be used to predict the behaviour of wild seals, so it is essential that the model can be applied across a range of individuals. We tested this by training the data on 10 random seals and then testing the model on two seals previously unseen by the model. While the cross-validation accuracy was lower than the training accuracy, we were still able to classify the seals behaviour well with some of the models. Previously, the effect of individual has been shown to have a large influence on the overall accuracy of the model [48]. Fitting a model to an individual generally causes it to over-fit, thereby losing the generalisability of the model. By including many different animals of different sizes, and testing it on two animals previously unseen by the model, we will be able to use the best model to predict the behaviours of many otariids. However, it is uncertain whether this model could be used with other pinniped species. For example, the very different gaits of the phocids in water and on land would likely influence the overall predictive ability of the model [60].

### Influence of feature statistics (characteristics)

We chose characteristics that could easily be determined from animals tagged in the wild to test how they would influence the overall accuracy of the models. We found that by including these variables (place, age, sex, species, mass and accelerometer attachment method) that the models training and testing accuracies improved.

The individuals in this study differed in age, sex, species and mass, which we hypothesised to influence model accuracy. Previously it has been shown that with dogs there were no differences behaviour prediction in inter-breed comparisons [46]. It is suggested that the lack of difference in body morphology would explain the lack of difference. Here we suggest that including these types of information in the model can help improve accuracies. Sea lions as a class differ from fur seals in several aspects of body locomotion, and allowing the model to distinguish between the two might explain some of the model improvement [61]. It may also be explained by differences in prey processing tactics that we observed the species using [62], as this type of behaviour was not examined in the dogs. Specifically, sea lions can process prey with their fore-flippers and chew their food, a phenomenon not observed in fur seals [62]. By including these details in the model we were able to improve training accuracy by between 5.3 and 7.8% cross-validation accuracy by between 5.3 and 20.1%. Considering we would know these characteristics of wild seals it is a worthwhile endeavour to include these features in models.

### Conclusions

The aim of this research was to determine the optimum method of automatically classifying many behaviours of a highly dynamic animal living in a complex environment using an accelerometer. Due to the large number of behaviours that animals can display, we further sought to investigate whether behaviours could be grouped for simpler prediction. Classifying behaviours of an animal is extremely difficult, and despite having captive animals under command we were still unable to capture all behaviours. Of the behaviours we did capture we were only able to classify three of the groups of behaviour with relatively high accuracy (travelling had poor accuracy results).

These results are important for the application of accelerometers to wild animals. When using supervised machine learning to classify behaviour it is likely that the animal will display behaviours that have not been trained into the algorithm. Therefore, the model will do its best to fit it into a group that is the most representative. For models that have been trained on a few select behaviours, this means there will be a significant amount of time that the animals mode of activity will be misclassified, leading to inaccurate activity budgets (if that is indeed the goal of the research). For example, the poor result for classifying travelling in our study means that for around half the time that the seal is travelling, they will likely be classified as grooming or foraging.

These models are complex and need to be treated as such. Providing a model with many repeats (hundreds if possible) of highly diverse behaviours in a related environment is vital to being able to use this technology and these models on wild animals. Though, this still does not guarantee that the behaviours observed from captive animals will directly translate to their wild counterparts. The environment in which behaviours were observed (captivity) is incredibly different to the wild. Small pools, dead prey and human instruction may alter the way that animals display behaviour. In particular we were unable to replicate prey chasing in captivity which would have helped to differentiate between travelling and foraging. Captive surrogates have been used successfully to train models with vultures [17] and when developing models from the same species an over 90% accuracy rate can be obtained [47]

Applications of this type of behavioural analysis include developing time-energy budgets of free living seals. To estimate energy expenditure in the field the durations of different activities are multiplied by their corresponding energetic cost [63]. Ethograms developed from accelerometers provide the essential information of time spent in various activities, and using accelerometers energy expenditure can be estimated concurrently [56]. Further, these types of models can be used to monitor populations of animals over time. For example, knowing how much time animals spend foraging between years can be indicative of the prey availability and can identify the potential vulnerability within groups [64].

## Supporting Information

S1 File Description and acceleration profile for 26 unique behaviours recorded. Black line–x axis acceleration; grey line–y axis acceleration; orange line–z axis acceleration.
(PDF)
