# Supplementary material for: Seeing It All: Evaluating Supervised Machine Learning Methods for the Classification of Diverse Otariid Behaviours
Source: PLoS One. 2016 Dec 21;11(12):e0166898. doi: 10.1371/journal.pone.0166898 (PMC5176164; doi:10.1371/journal.pone.0166898)
Supplement: S1 File — Black line–x axis acceleration; grey line–y axis acceleration; orange line–z axis acceleration. (PDF) [file pone.0166898.s001.pdf]

S1 File. Description and acceleration profile for 26 unique behaviours recorded. Black line – x axis acceleration; grey line – y axis acceleration; orange line – z axis acceleration.

Behaviour	Behaviour description	
RESTING		
Lying	Full body contact to the ground with minimal movement	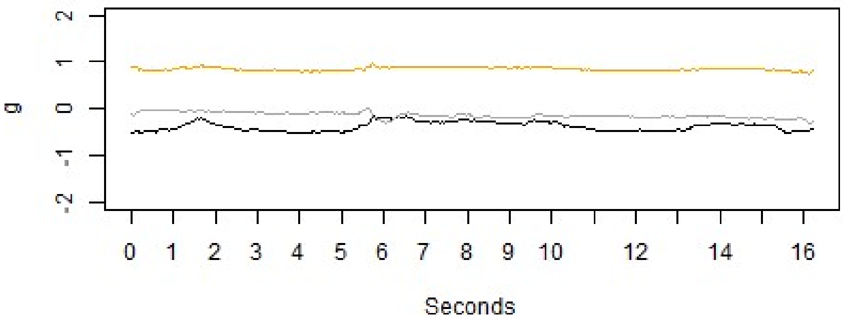
Sitting	Perched on fore-flippers with rear on ground, head up and alert with minimal movement	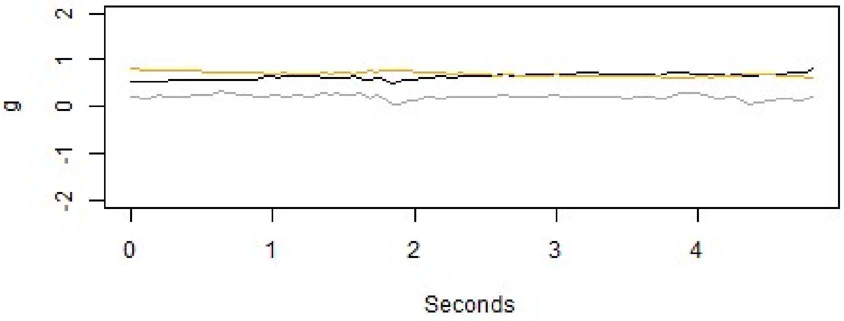

Behaviour	Behaviour description	
Still	Floating at the surface or beneath the surface of the water with no movement	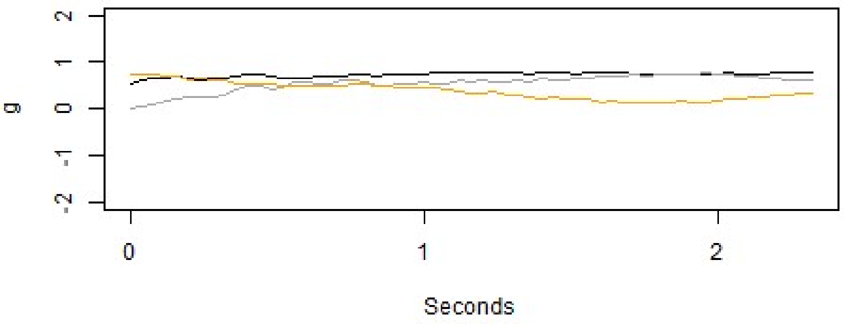
GROOMING		
Scratch	Perched on fore-flippers with rear on ground, head down towards rear and one rear flipper scratching neck or head	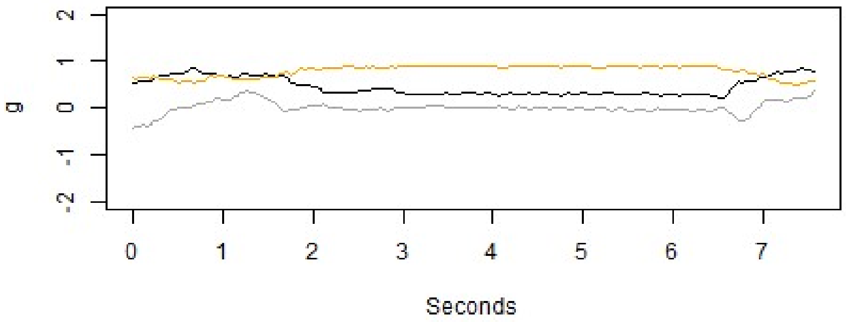

Behaviour	Behaviour description	
Rubbing	Using the fore-flipper to rub the fur on the rump	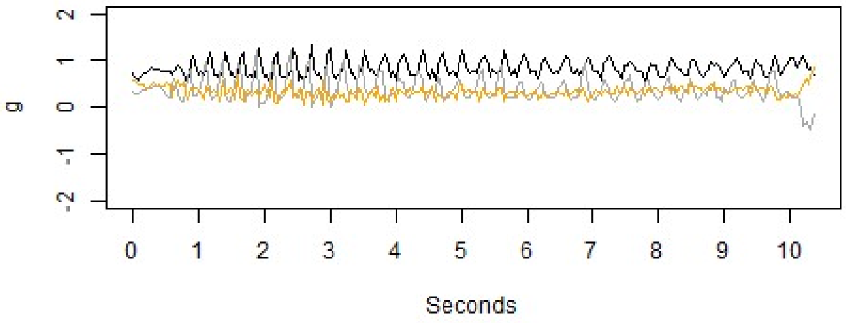
Sailing	Lie vertically in the water with one flipper in the air	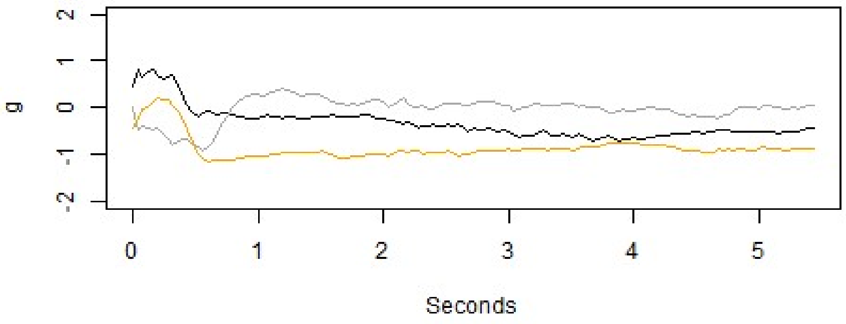

Jugging

Hanging upside down in water with hind flippers in the air

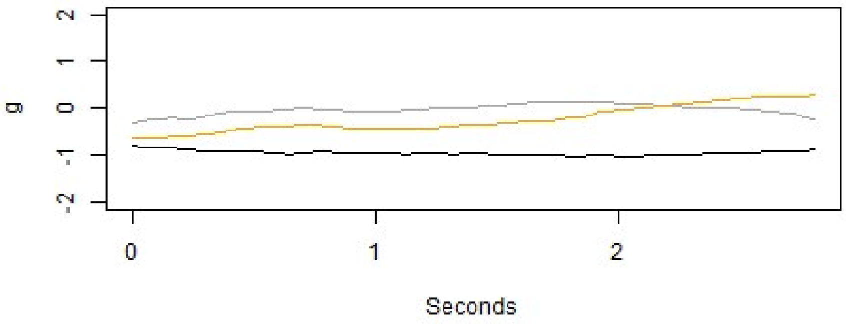

Behaviour	Behaviour description

Facerub

Using fore-flippers to rub face and whiskers

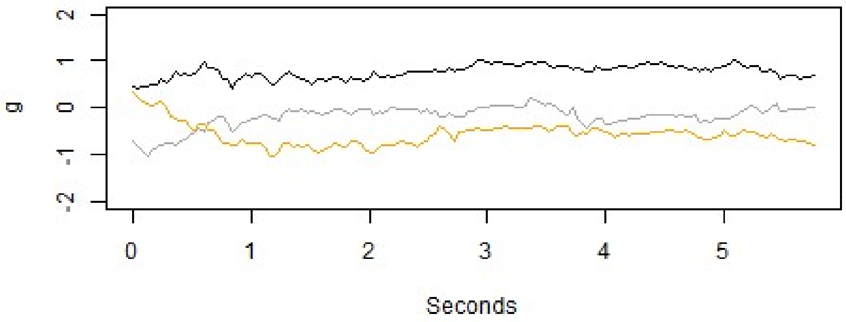

Shake	Short and sharp movement of the head left and right to remove water from the fur	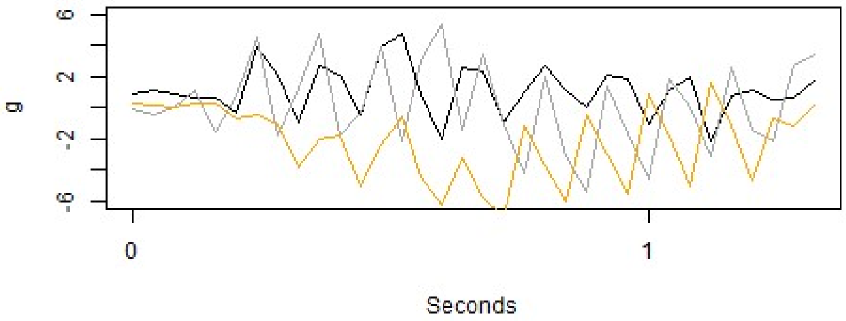
Rolling	Rolling at the surface of the water and rubbing fur with the flippers (Liwang 2010)	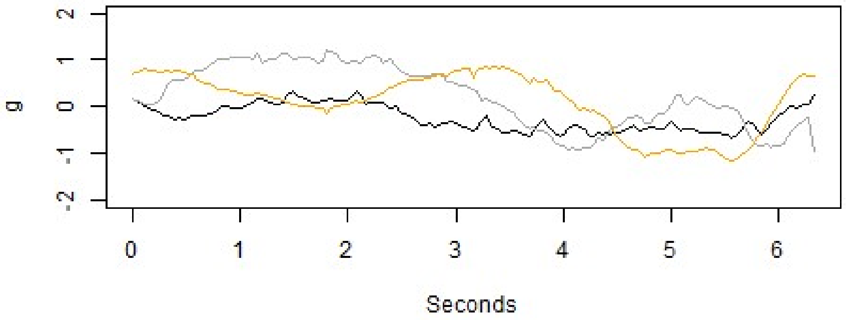

Behaviour	Behaviour description	
TRAVELLING		
Moving	Locomotion out of water, four flippers used as legs, stomach does not touch the ground	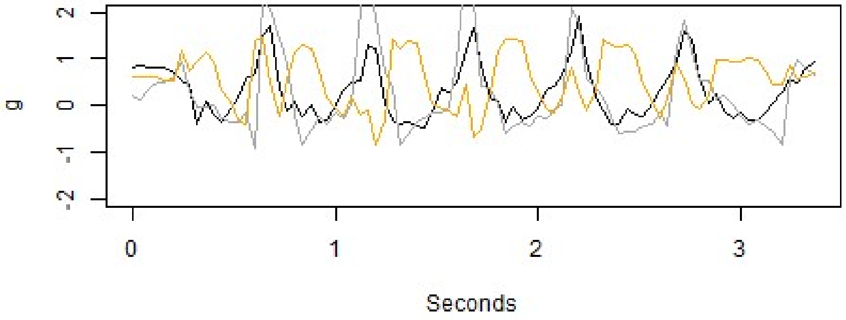

Slow	Locomotion at the surface of the water, using fore-flippers to move forward	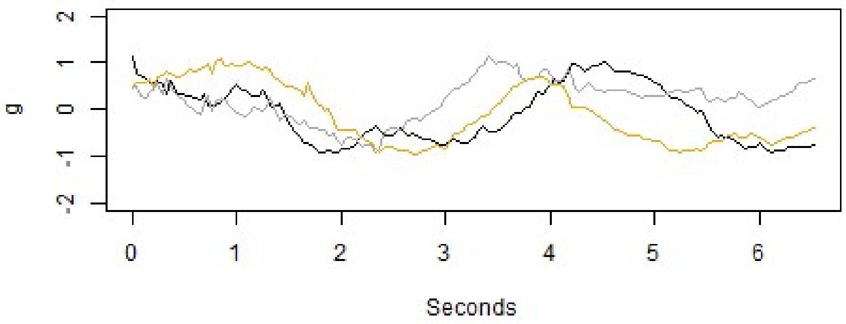
Behaviour	Behaviour description	
Swimming	Locomotion below the surface of the water using front flippers for propulsion	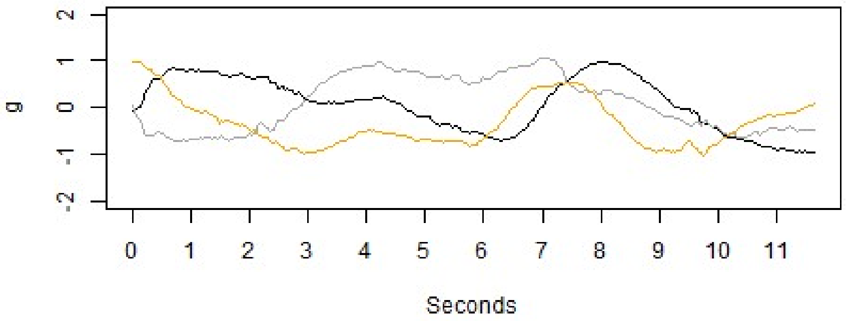
Fast	Rigorous propulsion of the fore-flippers at the surface of the water to accelerate (preceded by a small jump and followed by a glide)	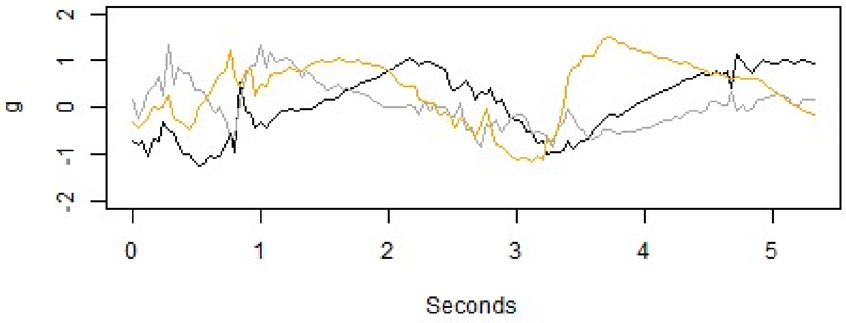

Porpoising	Fast swimming near the surface accompanied by parabolic leaps (Au and Weihs, 1980)	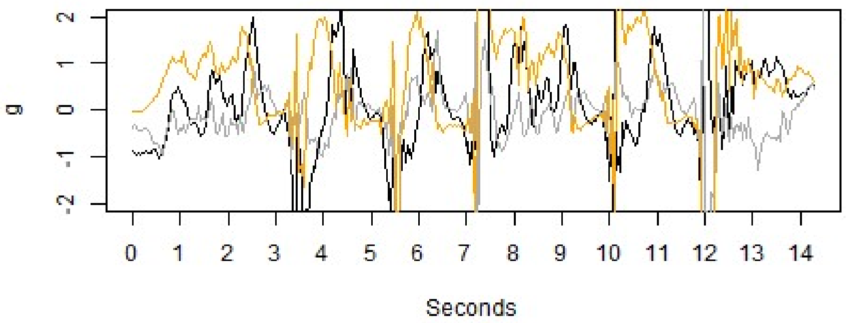
**Behaviour**	**Behaviour description**	
FEEDING		
Chewing	large head movement at the surface of the water to break food	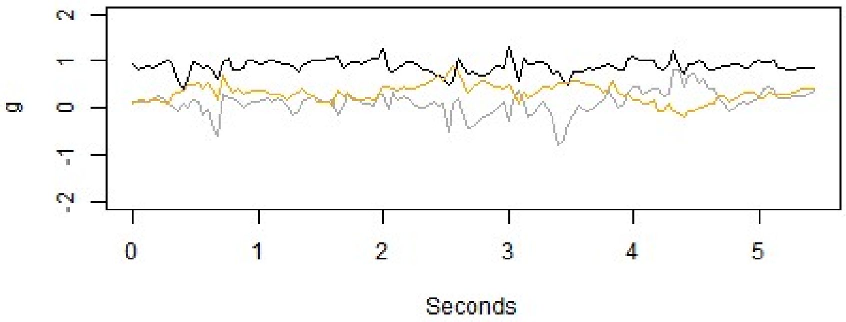
Searching	Locomotion below 1m actively searching for or manipulating food	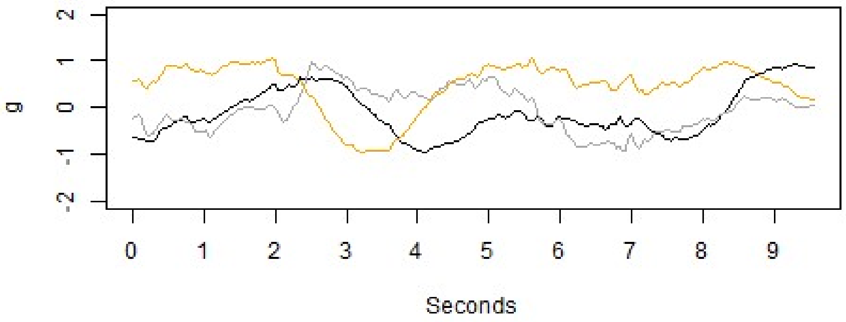

Behaviour	Behaviour description	
Capture	Head strike to capture food from the water column while swimming	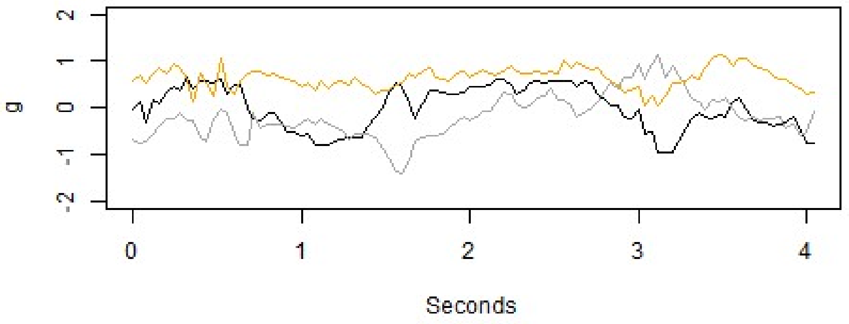
Thrash	Quick movement of the head back and forth while holding a prey item between teeth Elongated movement of the head back and forth with the purpose of breaking prey	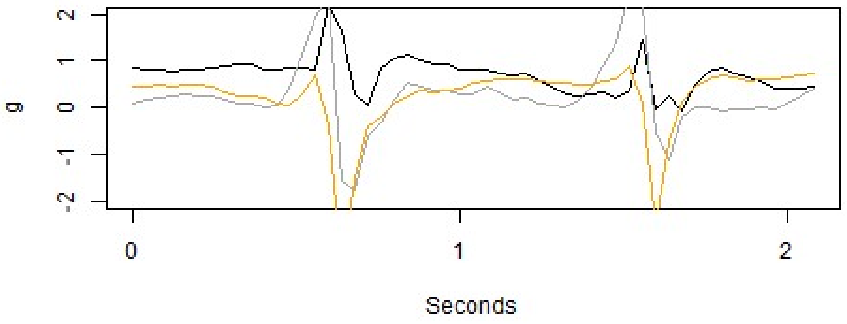
Manipulation [prey handling]	moving prey at the surface of the water (>1m) to manipulate into the best position for consumption	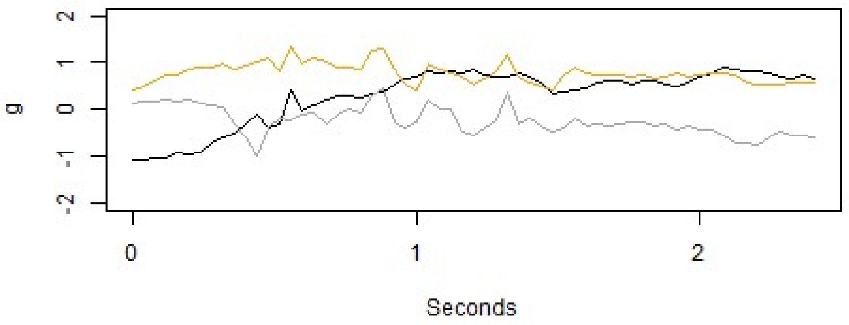

Behaviour	Behaviour description	
Holdntear	Grip prey between the fore-flippers in an upright position and use teeth to tear pieces of fish from the body	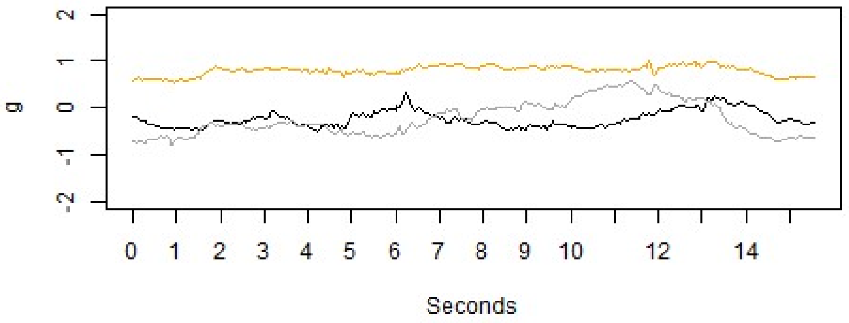
More	Description	
Playing	Rapid movements when interacting with other seals	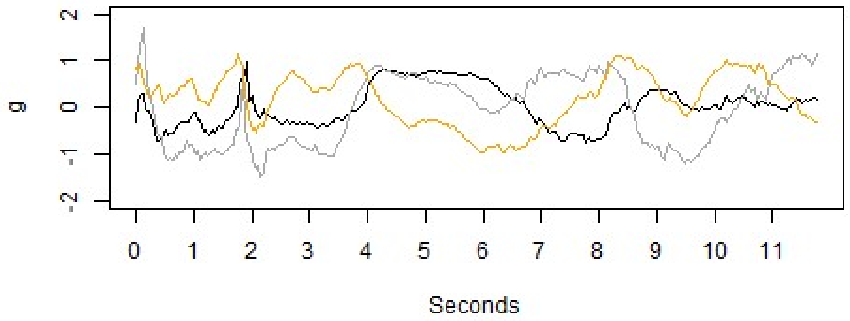
Other	in/out/jump/eating	
